# Do nursing homes for older people have the support they need to provide end-of-life care? A mixed methods enquiry in England

**DOI:** 10.1177/0269216310387964

**Published:** 2011-03

**Authors:** Jane E Seymour, Arun Kumar, Katherine Froggatt

**Affiliations:** The Sue Ryder Care Centre for the Study of Supportive, Palliative and End of Life Care, School of Nursing, Midwifery and Physiotherapy, University of Nottingham, UK; The Sue Ryder Care Centre for the Study of Supportive, Palliative and End of Life Care, School of Nursing, Midwifery and Physiotherapy, University of Nottingham, UK; The International Observatory on End of Life Care, Institute for Health Research, Lancaster University, UK

**Keywords:** Case studies, end-of-life care, mixed methods, nursing homes, older people, survey

## Abstract

Nursing homes are a common site of death, but older residents receive variable quality of end-of-life care. We used a mixed methods design to identify external influences on the quality of end-of-life care in nursing homes. Two qualitative case studies were conducted and a postal survey of 180 nursing homes surrounding the case study sites. In the case studies, qualitative interviews were held with seven members of nursing home staff and 10 external staff. Problems in accessing support for end-of-life care reported in the survey included variable support by general practitioners (GPs), reluctance among GPs to prescribe appropriate medication, lack of support from other agencies, lack of out of hours support, cost of syringe drivers and lack of access to training. Most care homes were implementing a care pathway. Those that were not rated their end-of-life care as in need of improvement or as average. The case studies suggest that critical factors in improving end-of-life care in nursing homes include developing clinical leadership, developing relationships with GPs, the support of ‘key’ external advocates and leverage of additional resources by adoption of care pathway tools.

## Introduction

Nursing homes are an increasingly common site of death. In England, 16% of all deaths take place in the long-term care sector,^1^ with most occurring in nursing homes among over 85 year olds.^2^ Predicted socio demographic trends show rapid increases in the numbers of people aged over 85 and of single households, with concomitant decreases in the availability of informal carers. These trends mean that nursing homes are likely to remain as important sites of end-of-life care for the foreseeable future.^3^

Older people admitted to nursing homes have been estimated to have a life expectancy of 9–12 months,^4^ with those who have dementia having the shortest life expectancy.^5^ However, the complexity of chronic and co morbid conditions^6^ among residents makes it difficult to recognize and manage the terminal phase.^7^ Many residents die after a period of diffuse deterioration marked by increasing disability and frailty,^8^ rather than a clearly identifiable ‘terminal illness’.^9^ There is evidence that older people residing in care homes receive variable quality in terms of both continuing ‘chronic’ disease care and end-of-life care because of clinical and organizational factors.^10^ One study in England^11^ has shown that 47% of homes have no provision for chronic disease management for care home residents, such as rehabilitation or physiotherapy, and others have only minimal levels, even though many residents could benefit from the latter. General practitioner (GP) services to care homes are not always organized optimally because of poorly defined funding for the provision of medical care.^12^ Similarly, input from clinical nurse specialists or palliative medicine clinicians is rare and, where it occurs, is reactive to crisis situations.^13^ Pain and symptom control is often poor as a result,^14^ and there is some evidence of inappropriate medication.^10,15^ Surveys of bereaved carers show high levels of dissatisfaction with end-of-life care in care homes.^16,17^

There has been a proliferation of different care home organizations across England, which creates challenges for the commissioning and funding of end-of-life care services in care homes.^18^ In addition, residents’ care is likely to be funded by a mixture of National Health Service (NHS), local authority and private monies, which is likely to make rapid access to resources difficult.^19^ Relationships between the range of health and social care agencies that intersect with care homes make care planning complex, and can lead to conflict in terms of the management of a resident’s final illness. This may increase the likelihood that some residents are admitted to hospital at the very end of life who might otherwise be supported in the care home setting.^20^

**Figure 1.**
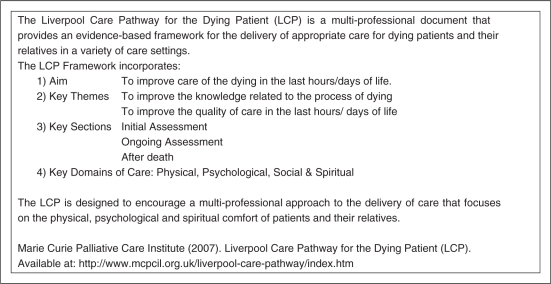
The Liverpool Care Pathway for the Dying.

**Figure 2.**
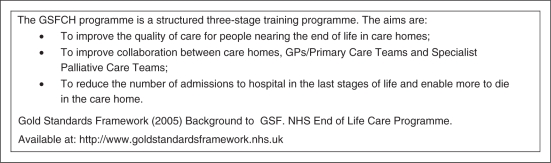
The Gold Standards Framework in Care Homes Programme (GSFCH).

**Figure 3.**
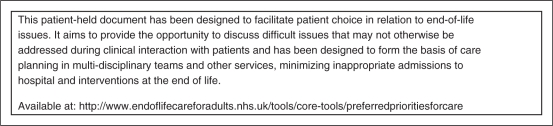
Preferred Priorities for Care (PPC) plan.

In England, a range of developments have occurred over the last 15 years to support the provision of end-of-life care in care homes. Specialist palliative care provision has been supported through the work of: (i) Clinical Nurse Specialists; (ii) the establishment of ‘hospice beds’ in nursing homes; (iii) the provision of palliative care education and training for care home staff; and (iv) the development of link nurse schemes.^20^ The promotion of general palliative care for any resident is now the main focus of developments,^21^ as reflected in the emphases of the National End of Life Care Programme, which has a specific stream of work concerning care homes.^22,23^ Within this, a number of initiatives are being promoted to support the provision of end-of-life care in care homes, including the care and service planning tools: the Liverpool Care Pathway (LCP) for the Dying (Figure 1); the Gold Standards Framework (GSF; Figure 2); and the Preferred Priorities for Care (Figure 3). There are also a large number of local initiatives and developments.^23^

This paper draws from a study commissioned by the National End of Life Care Programme in England^24^ in the context of the programme’s attempt to assess the effectiveness of the various initiatives outlined above and to inform recommendations for the further development of policy and practice. The aim of the study was to identify key factors in the wider health and social care system influencing the quality of end-of-life care provided in nursing homes. The study aim and method of enquiry was informed by an expert steering group made up of representatives from the funders, umbrella organizations for nursing home and care providers, regulatory bodies and NHS Trusts.

## Methods

### Setting and design

A mixed methods design was employed, consisting of two in-depth qualitative case studies^25^ of nursing homes and a postal survey of the managers of 180 nursing homes surrounding the case study sites. Names and addresses of care homes with nursing care and registered to care for ‘old age’ (not falling into any other category) were identified from the Commission for Social Care Inspection (CSCI; now the Care Quality Commission) database,^26^ which is the regulatory body for care homes in England. The survey was developed from a review of previous surveys used in related research^27,28^ and piloted in two focus groups with care home staff. The survey included questions about the profile of deaths in the homes, access to external support and barriers to and perceived priorities for improving end-of-life care.

The case study homes were in Northern England. One was in a rural area and one in a city. They were purposively selected as ‘instrumental’ case studies^25^ to facilitate understanding of a wider set of issues following consultation with colleagues from the National End of Life Care Programme and local stakeholders in end-of-life care practice and policy. The homes were known to provide a good standard of end-of-life care (we assessed this via reports from stakeholders and from homes inspection reports, which are accessible on the database of the CSCI) and our focus was on how key staff experienced some of the issues found in the survey and to see how the homes had addressed commonly perceived and encountered problems. Qualitative interviews were held with seven members of care home staff and 10 ‘stakeholders’ nominated by the staff as providing them with external support. Details of interview participants are shown in Table 1*.*

**Table 1.** Interviews conducted in each case study

		‘City’ care home	‘Rural’ care home
Inside the care home	Internal interview 1	Care Home Manager	Care Home Manager
	Internal interview 2	Assistant Manager	Assistant Manager
	Internal interview 3	Lead Nurse	Lead Senior Carer
	Internal interview 4	Hobbies and Activities Coordinator	
Outside the care	External interview 1	PCT Education Facilitator	PCT End of Life Care Pathway Facilitator
home	External interview 2	Lead commissioner for continuing care	Community Matron
	External interview 3	General Practitioner	Community Psychiatric Nurse
	External interview 4	PCT Liverpool Care Pathway facilitator	Local Care Home Manager
	External interview 5		General Practitioner
	External interview 6		Community and Hospital Macmillan Nurse

### Analysis

Survey data were analysed to produce descriptive statistics with the aid of the Statistical Package for the Social Sciences (SPSS)^©^. Free text comments added to the survey document were subject to content analysis. The findings from the survey were used to design an aide memoire and initial coding frame for the qualitative interview data. The framework was then modified to include any new issues within interviewees’ accounts. In addition to a thematic analysis of the interviews, we sought to understand the narratives recounted about the recent history of the care homes and developed short historical profiles of each home, focusing on understanding how problems associated with external support for end-of-life care had been addressed. One researcher (AK) conducted the initial analysis, which was then checked by JS and KF. Summary reports of the project were sent to each care home that had participated in the study. Case study care homes provided comments on a draft of their case study report, clarifying points of detail and weight of interpretation. This acted as a means of respondent validation.^29^

### Ethical review

Ethical committee approval was gained through the UK National Research Ethics Service. We gained research governance approval to interview stakeholders in the case studies from relevant Primary Health Care Trusts.

## Findings

### Survey

Following one reminder, we received a response from 46% (82) of the nursing homes surveyed.

#### Profile of deaths, self-rating of end-of-life care and use of ‘pathways’

Seventy-four percent (*n* = 62) of the care homes that returned the survey reported that they were registered as both residential and nursing homes; the remaining provided nursing care only. Responding homes ranged in size from 19 to 180 beds and reported a mean of 18 deaths per home (range 2–90) in 2007. Of the 1182 residents’ deaths reported, 76.5% (904) took place in the home, 23.3% (275) in hospital and 0.25% (3) in a hospice. Seventy-seven percent of deaths were reported to be caused primarily by non-cancer conditions.

Seventy eight percent of the responding care homes (64) self-rated the quality of their end-of-life care. Of these, one home described it as ‘needs improving’, three as ‘average’, 33 as ‘good’ and 27 as ‘excellent’. Ninety-eight percent (80) of the responding care homes responded to a question about use of end-of-life tools. Most (50) reported use of the LCP, with smaller numbers reporting use of the GSF (21) and/or Preferred Priorities of Care (PPC) (4). Sixteen homes were using both the LCP and the GSF. Eight care homes reported they were using their own care pathway or one that had been locally developed. Of the 60 homes rating their end-of-life care as ‘good’ or ‘excellent’, the majority (46) reported use of a care pathway. The four homes rating their care as ‘needs improving’ or ‘average’ reported that they were not using a care pathway.

#### Levels of support received

Care homes were asked to describe the level of support they received from a list of different agencies and to categorize their use of this support in the following terms: ‘not requested’, ‘not at all’, ‘only a little’, ‘some’ or ‘a lot’ (see Table 2). Seventy-two (*n* = 59) responded to this question. Of those, 97% (58) reported that they received ‘some’ or ‘a lot’ of support from GPs; 94% (58) ‘some’ or ‘a lot’ of support from family members. The majority reported similarly about specialist nurses (80%; 47), district nurses (51%; 30) and specialist palliative care teams (54%; 32). Less than half of homes reported receiving some or a lot of help from social workers (49%; 29), community matrons (20%; 12), NHS hospitals (31%; 18), staff from the National End of Life Care Programme (32%; 19) or the CSCI (29%; 17). A small minority of homes responding to this question rated voluntary organizations (9%; 5), support groups (13%; 8) or learning disability teams (7%; 4) as providing similar levels of support to them. Reported levels of support were consistent with whether or not homes reported that they requested help from an agency; noteworthy here is that one quarter of homes that responded to this question said that they did not request help from specialist palliative care teams (24%; 14).

**Table 2.** Reported levels of support received from external staff and agencies

	Not requested	Not at all	Only a little	Some	A lot
Social Worker	20% (12)	15% (9)	15% (9)	34% (20)	15% (9)
GP	0% (0)	0% (0)	2% (1)	22% (13)	76% (45)
Specialist nurses	3% (2)	5% (3)	12% (7)	36% (21)	44% (26)
District nurses	14% (8)	7% (4)	29% (17)	19% (11)	32% (19)
Community matron	39% (23)	24% (14)	17% (10)	12% (7)	8% (5)
Specialist palliative care teams	24% (14)	10% (6)	12% (7)	31% (18)	24% (14)
Learning disability team	68% (40)	20% (12)	5% (3)	5% (3)	2% (1)
CSCI	42% (25)	24% (14)	5% (3)	22% (13)	7% (4)
NHS Eolc programme staff	42% (25)	17% (10)	8% (5)	15% (9)	17% (10)
NHS Hospitals	37% (22)	8% (5)	24% (14)	24% (14)	7% (4)
Voluntary organizations	66% (39)	17% (10)	8% (5)	7% (4)	2% (1)
Volunteers	61% (36)	24% (14)	3% (2)	10% (6)	2% (1)
Family members	2% (1)	0% (0)	3% (2)	47% (28)	47% (28)
Support groups	53% (31)	15% (9)	19% (11)	10% (6)	3% (2)

The level of support received by care homes was further clarified by a question asking about whether visits by specific agencies were regular, occasional or infrequent/not requested. Table 3 shows details of reported frequency of visits from external professionals.

**Table 3.** Reported frequency of visits by external staff and agencies

	Regularly	Occasionally	Infrequently/not requested
	GPs	Community Macmillan nurses	Community matron
	District nurses	Macmillan nurses	Reflexologist
	Individual volunteers	Speech and language therapist	Consultant in palliative care
	Social workers	Community psychiatric nurse	Acupuncturist
Agencies	Chiropodist	Occupational therapist	
	Pharmacist	Dietetic service	
	Spiritual support	Physiotherapist	
	Activities co-ordinator	Geriatrician	
		Counsellor	

Sixty-six percent of responding care homes (53) reported that they accessed specialist palliative care support in the form of a direct advice line to the local hospice or Macmillan nurses. Ninety-three percent of responding care homes (76) reported a range of 1–11 GP practices with which they liaised (mean of five); and a range of 1–34 individual GPs with whom they liaised (mean of 12).

Ninety-four percent of care homes (77) completing the survey responded to a question about whether the support and equipment they received varied according to the illness of the resident. Fifty six percent (43) stated that this was the case. Free text comments appended to the questionnaire showed that support for residents with cancer was viewed as better than support given for those with dementia. Variability of support from GPs was frequently highlighted, as were issues of funding and organizational boundaries. Table 4 displays comments added to the questionnaire about support.

**Table 4.** Examples of qualitative comments about support received for end-of-life care

Residents’ needs and illness.	*‘Resident’s needs vary due to the nature of illness.’*
	*Care home no. 2-043*
	*‘The support increases as a resident’s illness progresses.’*
	*Care home no. 2-008*
	*‘Appears to be less support for older people and younger people suffering with dementia.’*
	*Care home no. 1-060*
	*‘Easier to access services for cancer patients who are terminally ill.’*
	*Care home no. 1-079*
GP support	*‘At times can vary re: GP Surgery/ individual GP's perception of individuals needs and nurse has to act as advocate.’*
	*Care home no. 2-001*
	*‘Depends whether patient's GP is in PCT X or PCT Y. X seems very efficient, Y often lacking.’*
	*Care home no. 2-022*
PCT boundaries and care home classification	*‘It varies on where they lived or are transferred from and how they are funded.’*
	*Care home no. 2-006*
	*‘If diagnosis made before admission there tends to be a better support network in place.’*
	*Care home no. 2-017*
	*‘Dependent upon the classification i.e. residential/nursing however our practice is to request review of care status and our mission is to ensure end-of-life care within the residents’ home.’*
	*Care home no. 1-063*
	*‘Residential - full resources. Nursing - care home has to provide.’*
	*Care home no. 1-087*

#### Barriers to end-of-life care

Care homes were asked to provide qualitative comments about any barriers that made it difficult for them to access support for end-of-life care. Sixty-one percent (34) provided comments and these related to GPs lack of support and reluctance to prescribe appropriate medication, lack of support from other agencies and community resources, out of hours support, cost of syringe drivers, lack of information about support and training available. Table 5 provides a summary of the qualitative comments made.

**Table 5.** Examples of qualitative comments about barriers to end-of-life care

Barriers in accessing support for end-of-life care	
GPs	*‘GPs not always keen to issue end-of-life drugs or to visit promptly’.*
	*Care home no. 1-021*
	*‘Some GPs do not appear to support end-of-life care.’*
	*Care home no. 1-057*
	*‘Lack of support from GPs with specialist or interest in palliative care.’*
	*Care home no. 2-030*
	*‘Some GP support better than other.’*
	*Care home no. 2-061*
Wider system	*‘Other agencies’ attitudes towards nursing homes. They appear to hold back when we discuss end-of-life care. Mainly hospital staff.’*
	*Care home no. 1-020*
	*‘Resources in the community.’*
	*Care home no. 1-064*
	*‘Delay in information being passed on to on call GP.’*
	*Care home no. 1-075*
	*‘Sometimes too many different people involved. I.e. Community Matron, GP liaison, NHS, making communication difficult at times.’*
	*Care home no. 1-087*
	*‘Family expectations – Prejudice from Hospital, GPs, and Social Workers.’*
	*Care home no. 1-013*
Lack of information/resources	*‘Access to resources - particularly for items not on prescription.’*
	*Care home no. 1-041*
	*‘Lack of information about services available and how to access.’*
	*Care home no. 2-001*
	*‘Financial - Cost of syringe drivers.’*
	*Care home no. 2-006*
	*‘Not enough phone numbers available.’*
	*Care home no. 2-010*
	*‘Training - difficult to get onto syringe driver training’.*
	*Care home no. 2-037*
	*‘I feel the training for GSF is limited; there is a waiting list for training which is a huge disadvantage for homes nursing people at the end of life.’*
	*Care home no. 1-079*
	*‘To help staff plan end-of-life care, we would benefit from up to date information regarding residents past treatment from hospitals, clinics, etc. We are dependent on information from GPs, they don't always think that we need this information but it is vital.’*
	*Care home no. 2-030*
Out of hours	*‘Clients end-of-life wishes not taken into account by some out of hours staff.’*
	*Care home no. 2-029*
	*‘Friday afternoon discharges/slower system at weekends’.*
	*Care home no. 2-055*
	*‘Getting medications for LCP at night/Weekends/Bank Holidays.’*
	*Care home no. 2-085*
	*‘Evening/weekend support can be very patchy.’*
	*Care home no. 2-086*
	*‘No late night pharmacy support.’*
	*Care home no. 1-075*

The case studies were conducted to shed light on how issues revealed by the survey were encountered in contrasting rural and urban contexts, by two homes judged by expert stakeholders to provide a good quality of end-of-life care.

### Case studies

Both the nursing homes studied as cases had very different contexts and conditions in which they were trying to develop their practice and for both, end-of-life care was only one aspect of their work. However, both were remarkably similar in terms of the ability demonstrated by senior staff to show leadership and to engender aspirations for continual improvement of standards of end-of-life care. Both homes were actively engaged in networking with other homes in their locality, albeit at different stages of development, and both were fully aware of the range of problematic issues in relation to external support: one home had largely resolved many of the problems, while for the second home, this was very much ‘work in progress’. Below we provide sketches to show how the two case study care homes had contrasting experiences in terms of external support for end-of-life care provision and were at different stages of progress. These sketches are composites of the analysis of qualitative interview data and we indicate the source of the data using superscript letters as indicated. In addition, Table 6 provides a comparison of the case study homes, showing how structural, internal and external factors related and influenced end-of-life care provision. Table 7 provides some exemplar quotes specifically relating to the experience of external support.

**Table 6.** Key findings from the case studies

‘City’ home	‘Rural’ home
*Structural conditions allowing development of end-of-life care practice*	*Structural conditions allowing development of end-of-life care practice*
Tendered for and won a contract for the provision of continuing and intermediate care in 2003	Implemented the Liverpool Care Pathway in 2006, following an attempt by the care home manager to seek out a method of ‘smoothing’ standards of end-of-life care
The first nursing home in the PCT to implement the Liverpool Care Pathway, in 2004	A community matron comes into post in 2006 with a remit to support care homes
Joined the National Gold Standards Framework (GSF) Programme for Care Homes in 2005	A community mental health care nurse comes into post in 2007 and supports the community matron in the care home work
*Intrinsic factors influencing quality of end-of-life care*	*Intrinsic factors influencing quality of end-of-life care*
A distinct philosophy of palliative and end-of-life care, strengthened by co staffing across the care home and continuing/ intermediate care unit and use of the pathways	An emerging philosophy of end-of-life care and clear aspirations for developing practice in end-of-life care. LCP seen as enabling this
Senior staff, who were in receipt of a palliative care certificate from the local hospice, showed leadership to others within and outwith the home	Leadership shown by senior staff in implementing the LCP and addressing problems in accessing extrinsic support
Learning and resource room in the home for use by all staff	Problems experienced in accessing training and education, especially where provided by the NHS. Staff paying and attending in own time
Shared emphasis on developing networks of communication with staff, key stakeholders, residents and relatives	Culture of good communication and regular staff meetings
Perceived support from care home owners, which has allowed relatively high staff–resident ratio	Perceived support from care home owners, which has allowed relatively high staff–resident ratio
Workforce perceived to be moderately stable and morale high	Workforce perceived to be moderately stable and morale high
Little reliance on district nurses	Some reliance on district nurses, who were a scarce resource in the locality with no clear remit to attend nursing homes
*Extrinsic factors influencing quality of end-of-life care*	*Extrinsic factors influencing quality of end-of-life care*
Staff invited to attend multidisciplinary meetings in the PCT relating to the GSF and palliative and supportive strategy more broadly	Care home staff not attending multi-disciplinary team meetings and felt relatively isolated from wider end-of-life care practice in the PCT
Links with and support from with GPs and Macmillan nursing services has improved as end-of-life care practice in house has developed. This has begun to resolve some medical staffing, prescribing and ‘out of hours’ problems	GP support has been problematic in the past and is still variable. Out of hours support perceived as inadequate
Well supported by key PCT staff and an informed commissioner	Well supported by key staff, especially community matron and community mental health nurse. Macmillan nursing only accessed for cancer patients. Perceived threat of non-continuity of key roles in the PCT
Selected to host a syringe driver library for use by other care homes. Funded by a Big Lottery Grant, gained by the LCP facilitator	Ongoing struggles to gain syringe driver access. Partially solved by purchase of one driver by the PCT for use by local homes
Networking with other care homes is well developed	Networking with other care homes is under development

**Table 7.** Examples of interview quotes about external support accessed by the case study homes

Influence of ‘pathways’
*Staff within the Home felt that the care that we were providing although was good to the best of our abilities did vary. So depending how experienced the nurse was or how well they knew that person or how good their rapport, empathy, recognition of symptoms was that their care that you then provided would vary* …* we wanted to provide a better standard of care and the LCP seemed a way of providing that standard of care and actually, not exactly standardising but promoting the nurses’ [care] regardless of how much knowledge and experience they had -to actually pre-empt problems rather than allow them to run on.*
Care home manager observing the role of the LCP in end-of-life care, Rural case study
Access to ‘out of hours’ medication and syringe drivers
*So I’m trying, I’m working with our community pharmacist to try and see if there’s any way we can get named nurses in the nursing homes, when our district nurses are going to be issued with a box containing all the anticipatory drugs as well as the syringe drivers* …*.We don’t know [when this system will start]* …* it’s like everything else it’s not, it doesn’t happen overnight* …*.*
Community matron describing issues in access to out of hours medication, Rural case study
*I had a patient down in intermediate care, which is where I was working, and she needed, over a Bank Holiday weekend, she needed a syringe driver and she needed something like the Pathway that we didn’t have at the time. And she begged me not to send her into hospital and she ended up going to the hospice because we hadn’t a clue where to get a syringe driver from. The doctor didn’t know anything about the drugs or what we should be using. It was the day before Good Friday, which was a long Bank Holiday weekend, and that lady died at [the local hospice] 48 hours later with staff that she didn’t know and she didn’t want to go. And I think it’s from then I decided that I didn’t want this to happen again to anybody and that we needed to sort it out here so that even if it was just our home we actually knew where we were going and where to get the equipment from.*
Care home manager describing a pivotal experience, City case study
Relationships with GPs
*I think it [the GP and nursing home relationship] is excellent with [Rural care home]. It’s not quite so good with others I don’t think. I think we get much poorer communication and the information sharing’s not as good. So sometimes you go and nobody seems to know why you’ve been called or what’s going on or, you just get the impression that nobody really knows, you know, has got a particular handle of what’s going on in certain patients* …* I think just to make sure that, you know, you have all the information to hand on both sides really. Because, you know, there have been occasions where they’ve passed on a message here and it hasn’t necessarily got through to us and then you go and you’re not quite sure what you’re looking for.*
GP explaining why relationships with care homes are sometimes strained, Rural case study
…*Very often the question of end-of-life pathway drugs has been brought to us by the nursing staff [here] and always appropriately, as far as I’m concerned* …* I mean we’re probably more familiar to them here than any other practices because we spend so much time here. So I think that helps really because the more you know people the more you come to trust them, or you could put it the other way, I suppose.*
GP’s view of the importance of mutual trust, City case study
*One or two GPs I think sometimes may be less geared up to end-of-life care than others. So I think that’s a challenge for the staff and we are working on that. I mean, generally, I think things have improved a great deal but there are just a few GPs that do hold back probably more than others* …* I think whether they [don’t] know the patient very well, whether they’re aware of the drugs they need to be prescribing, time probably as well, you know.*
Macmillan nurse observing variable practice among GPs, Rural case study
Support from district nurses
*The district nurses don’t like to come into nursing homes to do jobs that they think that we should do. Which sounds quite awful but it’s doubling up essentially. They come into residential homes to give injections and change dressings but they don’t tend to come into nursing homes. The only reason they’d come into nursing homes is to give ‘flu vaccinations and things like that but then that tends to be the Practice Nurse from the surgery.*
Care home manager describing access issues with district nurses, Rural case study
Leverage of additional resources through ‘key’ contacts
*Because we work as part of a team, so we have access to physio, OT [Occupational Therapy], speech and language, diabetes specialist nurses, respiratory specialist nurses, and we also have a CPN [Community Psychiatric Nurse] who works with me in the care homes and we have two healthcare support workers that we can put to support the care homes.*
Community matron listing support that can be levered for care homes, Rural case study
*People in care homes are the most vulnerable, or one of the most vulnerable of our population. They tend to have more healthcare needs than a lot of people in the community. Yet again, historically, we commissioned the service and then walked away.* …* I sensed when I came into post a lot of frustration that care homes often knew that there were services out there but they couldn’t access them. So in [the PCT] we’ve worked, and it’s a team of people that have worked really hard to break down those barriers* …*.we look at how the PCT can support the providers in that care home to deliver the best service that they can. It’s a real team effort.*
Lead commissioner for continuing care, talking about ensuring access to PCT resources, City case study
Access to training and education
*I think there should be more training about palliative care and end-of-life care than there is at the moment and I think that it’s quite difficult to access it or to hear about it. We’re a private nursing home so NHS courses, sometimes we hear about sometimes we don’t. If we do hear about it we have to pay to go on them which isn’t a problem but quite often you don’t get to hear about them* …*There aren’t enough courses to promote best practice.*
Deputy Sister observing lack of access to training opportunities, Rural case study

#### The ‘City’ care home

(Code for Interviews: A: care home manager; B: assistant manager; C: lead nurse; D: education facilitator; E: lead commissioner for continuing care; F: GP; G: LCP facilitator.)

The City care home was providing care to 58 residents at the time of the study. It had an integral unit for the delivery of intermediate and continuing care. Between 1 January and 31 December 2007, there were 30 residents who died in the home and five residents who died at the local hospital^c^. The CSCI completed an unannounced inspection of the home in December 2006 and found the atmosphere within the home as ‘welcoming and warm’. The ‘staff and residents spoken to had a sense of humour and appeared relaxed and comfortable’. The report described the ‘communication skills of the staff with individuals [was observed] to be very positive’. The report further found ‘residents and visiting relatives said the staff were generally very kind, helpful and friendly’ and ‘the relationship between residents/relatives and staff appeared positive and the residents were treated with respect’ (Commission for Social Care Inspection (2006); Inspection report). The current manager came into post in 2001, having previously worked as a district nurse for 25 years^a^. She has a very clear vision for the development of palliative care in the home, strengthened by co staffing across the care home and continuing/intermediate care unit. She had encouraged senior staff and others to undertake training and education in this area and was able to access training events in the locality. She had developed a resource room for use by care home staff. Staff morale was high and turnover relatively low.

The City home was the first within its Primary Care Trust (PCT) to implement the LCP for the Dying and the GSF, securing, in the opinion of a key stakeholder, its role as a leader in end-of-life care practice among its ‘peer’ care homes^g^. This followed the development of a palliative care strategy across the local PCTs in 1998, which had been followed by some changes in commissioning practice. The subsequent implementation of the GSF in the City home followed intensive mentorship provided to the care home staff by the LCP facilitator^g^ and the attendance of care home senior staff at GSF meetings. The care home manager reported that as a result of these regular meetings it had been possible for the City home to build good networks and rapport both with other local care homes and with colleagues in general practice and in specialist palliative care. These in turn enabled them to make timely referrals when they needed help with residents’ end-of-life care needs^a,b,c^.

Over time City home staff have been able to build good rapport with local GP practices^a^. They have developed a relationship of trust with one GP in particular from whom they now regularly receive visits. The support given from this and other GPs in providing end-of-life care is now highly esteemed by the senior staff and is complemented by the care home’s long-standing relationship with a local pharmacist, which aids access to prescribed medication when requested ‘out of hours’^c^. The role of the City home in end-of-life care was supported by an enlightened commissioner for continuing care, who recognized the importance of supporting practice in care homes and aiding access to PCT resources to ensure good end-of-life care for residents^e^. In the view of the commissioner, collaborative work across the Primary Health Care Trust boundary was beginning to break down the isolation of care homes. As a result, discriminatory attitudes and practices, which meant that once individuals were admitted to care homes with nursing they tended to be regarded as no longer entitled to the services commissioned by the PCT, were changing^e^.

Following a grant from the Big Lottery Fund (http://www.biglotteryfund.org.uk/ (accessed 15 June 2010)) in 2007, the LCP facilitator explained how he was able to set up a ‘syringe driver library’ in the City home, which is for the use of care homes with nursing in the local area^g^. This had immediately resolved some, although not all, of the problems of access to syringe drivers among care homes in the locality. The City home was selected to hold the library because of its recognized expertise in end-of-life care.

#### The ‘Rural’ care home

(Code for interviews: H: care home manager; I: deputy sister; J: senior carer; K: LCP facilitator; L: community matron; M: community psychiatric nurse; N: local care home manager; O: GP; P: Macmillan nurse.)

The Rural home was providing care to 44 residents at the time of the study. Between 1 January and 31 December 2007, there were 25 deaths among residents, of whom 23 died in the home and two died in the local hospital^h^. The CSCI was involved in an unannounced inspection the home in April 2007 (Commission for Social Care Inspection (2007); Inspection report) and described the home as ‘domestic in character and well maintained’. The report further notes that there was: ‘ …a warm and welcoming atmosphere was evident on entering the home’ and ‘there was evidence that staff, service users and relatives have a good relationship and they chatted freely’, with service users and visitors having expressed that ‘care was at a good standard and staff were very kind and attentive’. The current care home manager has been in post for approximately four years, since 2004, and is a qualified Registered General Nurse (RGN). She had a clear view of the priorities for end-of-life care practice development in the home.

The Rural home implemented the LCP for the first time in 2006, following a deliberate attempt to seek knowledge about the pathway by the Care Home Manager. Its implementation was perceived to have ‘smoothed’ standards of end-of-life care, making them less dependent upon the particular skills and knowledge of staff or the attributes of their relationships with residents. However, unlike the City home, the Rural home encountered some significant problems in extending progress further, largely because of factors that lie outside of its control in the locality.

A key factor was perceived as the cessation of the contract for the end-of-life care facilitator within the PCT in 2008^h,l,m,p^. As a result, there is no longer a dedicated role within the PCT to introduce and provide ongoing training for the LCP. The manager of the Rural home also reported that accessing GP support, prescribed medication and transferring a resident to the hospital during out of hours was very difficult^h^. Some GPs endeavoured to overcome problems by ensuring medication was pre-emptively prescribed for individuals prior to the weekends, but this did not always provide the solution to unexpected problems among residents^h,j,o^. Lack of involvement in PCT meetings meant that staff in the Rural home felt isolated from wider end-of-life care developments in the locality^h,m,n^. This was improving since the appointment of a community matron with a remit for care homes and a community psychiatric nurse who supported the matron’s work with care homes.

The care home manager was keen that staff undertook training to develop their knowledge in end-of-life care issues, but found it difficult to access courses locally, since most of these were only available for NHS staff^h,j,k,m^. Those staff who did attend development events often did so in their own time and using their own funds. In spite of this, staff morale was high and turnover relatively low ^h,i,j,k^.

Accessing syringe drivers when these were needed was also a problem. The care home manager reported that the GP practice would be the first point of call for obtaining a syringe driver^h^. If the GP practice could not provide one then she would telephone the community matron or the local hospice and usually, by this lengthy process, would manage to obtain one^h^. However, gaining assistance with setting up the driver was sometimes problematic. The care home manager related a recent occasion when a driver was needed but she had not used one for over six months and no longer felt confident to set one up. After some difficulty, she managed to get advice and help from a district nurse^h^. Support from district nurses was generally perceived to be much needed but hard to access^h,i,j,o^. At the time of fieldwork, the community matron was trying to arrange for the care home manager to attend syringe driver training on a regular basis (potentially every three months) at the local hospice. It was reported that this was a means of pre-empting difficulties in using and calibrating syringe drivers, of reducing dependence on district nurses, and of strengthening the care home manager’s ability to cascade syringe driver training within the home^l,o^. By the end of the fieldwork, the PCT had loaned one syringe driver to the Rural home for them to use and to lend out to other care homes as required^l^.

## Study limitations

Since the study was small scale and exploratory, a decision was made to focus on homes registered to provide nursing care, as opposed to looking more generally at homes providing solely personal care (previously known in England as ‘residential care homes’). It is likely, however, that some of the issues reported here are also relevant to the latter. Furthermore, the homes we studied as ‘cases’ were not part of a wider ‘chain’ provider: this needs to be noted in making sense of the findings. There are a number of other limitations in the study. In keeping with other surveys of care homes, we had a relatively low response rate,^13,30^ which limits the representativeness of the findings. However, our response rate was considerably higher than that achieved by a National Audit Office survey of care homes on a similar theme and shows some similar findings.^30^ In the case studies, we were not able, for resource reasons, to access the views of older residents or their family carers about their experiences of care; nor, with one exception, were we able to gather the views of front line care assistant staff within care homes. Moreover, our purposive sampling of the two care homes with nursing that are the key case studies in the project meant that it was clearly in their interests to present the care that they provided in a largely positive light. However, by conducting a survey of other care homes in the localities of the cares home involved in the case studies, we have been able to contextualize the case study findings and thus enhance the validity of the study’s conclusions about the support that care homes need in order to provide appropriate end-of-life care. Furthermore, in their interviews with us staff were open and candid about the issues they faced in their daily work: they reported problems and ongoing challenges, as well as those things that were going well, communicating a sense of shared purpose in seeking to improve the capacity of care homes and build on the potential of care homes to provide excellent end-of-life care.

## Discussion

This paper has sought to examine the external influences on end-of-life care provision, reporting on how some issues from a simple survey to which 82 homes responded were manifest in two care homes selected as ‘instrumental’ case studies. It was conducted some 10 years after a seminal study in England, which showed how external influences are pivotal to the quality of palliative and end-of-life care provided in care homes for older people^31^ and took place at a time when the End of Life Care Strategy in England^32^ directed attention towards the need to improve end-of-life care in all settings and among all groups of patients in need.

The majority of nursing homes that took part in the survey reported some access to specialist palliative care services, such as the Macmillan nursing service and/or local hospice in the form of a 24-hour advice telephone line. This was supported by the experiences of the case study care homes both of which had some access to specialist palliative care support. However, in neither the case studies nor the survey did specialist palliative care support appear to be a regularly occurring feature of care provision. Rather, it was apparently dependent upon requests from the homes for such help. In the case of the City home, attendance at local palliative care and GSF meetings meant that they had developed networks of support that they could draw upon for end-of-life care issues. In addition, there is some evidence, from the survey data, of a lack of knowledge in nursing homes about available resources or about key staff who may be able to assist with end-of-life care, particularly for residents with needs arising from conditions other than cancer, such as dementia. It seems that external end-of-life care support provided to nursing homes, in most cases, is still predicated on a model of palliative care provision required for the classic ‘cancer’ trajectory, which is increasingly at odds with the reality of residents’ needs. Similar findings were found in a survey of clinical nurse specialists in palliative care, who reported that they tended to work only with cancer patients and in response to crisis situations in care homes.^12^ In the Rural home case study, it was clear that a major contribution to the ability of the home to cope with the residents’ needs was the recently instigated help they received from a community matron and a colleague with whom she worked closely, a community psychiatric nurse, both of whom had special responsibility for care homes in that area. The input the home received from these individuals was regular, proactive and planned, as opposed to irregular and crisis oriented.

The survey data indicated that most support with end-of-life care is provided to nursing homes by GPs and family members, while support from nurses external to the home was somewhat less marked. Neither of the case study care homes reported accessing district nursing support with any regularity; in the case of the City home, the existence of a continuing care unit meant that they could ‘cross cover’ for nursing needs and so perhaps no longer needed such support. In contrast, in the Rural home, district nursing support was a scare but much needed resource, particularly in relation to the management of syringe drivers. Where district nurse help had been received, it was in the knowledge that it was not an ‘allowable’ form of help, since no primary health care trust funds were available for the provision of additional nursing support to nursing homes. This issue of additional nursing support required by residents in nursing homes was highlighted by the Royal Commission on Long Term Care,^32^ and has never subsequently been resolved in the UK.

Support from GPs was clearly essential but was associated with a number of characteristic difficulties. Survey respondents briefly alluded in their qualitative comments to problems of communication, ‘out of hours’ coverage and variability in interest, skills and willing attendance to residents’ needs among the GPs they came into contact with: issues also reported in other studies.^30,34^ The case studies provided an opportunity to examine this issue from the point of view of two homes that had largely resolved problems with GP care. In the case of the City home, we heard how serious problems in the fairly recent past had been addressed by the development of collegial relationships nurtured by attendance of care home staff at local end-of-life care meetings. In the case of the Rural home, problems were still in evidence, but ameliorated by the development of mutual trust and understanding between one GP and the care home staff. The development of the latter was seen as essential by the two GPs whom we interviewed within the case studies; a condition of its development seemed to be in turn care home staff reaching a certain level of competence and expertise so that they could assess patients’ needs and appropriately refer patients to GPs.

The survey showed that nursing homes which implement an end-of-life care tool, such as the GSF or LCP, were more likely to describe their end-of-life care as ‘excellent’ and ‘good’. Providing some insight into how crucial the implementation of pathways was perceived to be among care home staff and their stakeholders, the case study data reveal how in each home implementation of the LCP, and, in the City home also the GSF, were seen as pivotal to end-of-life care improvement. Similar findings were found in a survey of care homes in England by the National Audit Office, although the validity of the findings from the latter are undermined by a very low response rate of below 10%.^30^ The relationship between the use of ‘tools’ and quality of end-of-life care has been identified as needing further examination^35^ in all care settings, including nursing homes. One worrying finding was the inconsistent access to education reported by the homes that responded to the survey and the case study homes: staff were often attending study days in their own time and using their own resources and found they were sometimes excluded from mainstream NHS provision. It is likely that the in-house education levered by participation in the LCP and GSF is critical, although whether the cost of participating in the GSF programme inhibits some homes from participation needs to be ascertained. Gibbs,^36^ in study of knowledge about pain management, noted how nurses in private nursing homes feel less skilled, are isolated and lack educational opportunities about ‘mainstream’ practice in palliative care. Under-treated pain remains a significant problem in care homes internationally.^37^

In the case study homes, clinical leadership and a reasonably stable workforce (supported by a good CSCI reports) meant that the homes had the capacity to successfully implement the tools and to provide some degree of ongoing education to staff. The role of clinical leadership in care homes has been identified as critical to care quality.^38^ The care managers in the case study homes were in turn supported by key individuals external to the care homes, such as GSF and LCP facilitators, who often were in a position to ‘lever’ additional resources. The importance of resource leverage and the role of nurses in the latter were similarly observed in a study of the outcomes of Macmillan Nursing conducted in the UK in the 1990s, but remains an under-researched issue in palliative and end-of-life care.^39^ In the case of the City home, the influence of a supportive lead commissioner for continuing care was also evident. This commissioner had been able to implement changes in commissioning practice through a process of participating in strategic developments directed at improving the co-ordination of palliative and end-of-life care in the wider vicinity. The City home particularly demonstrates the powerful synergy that can occur between factors such as small-scale practice innovation, personal aspirations, the provision of effective external links for networking and support and wider changes in commissioning practice and attitudes. These all coalesced in the City home, such that they had begun to overcome some intractable problems reported elsewhere. They are now in a position to provide support to other care homes in the locality and to be a ‘beacon’ of good practice. Questions need to be asked about those nursing homes that do not have such a fortunate set of circumstances: it was clear from the survey data that a minority of homes were excluded from the outside support that flows from participation in the LCP and GSF implementation process, where the latter become ‘enablers’ of practice development within the care home and levers of support outside it. Care must be taken to ensure that such isolation does not become a catalyst for the widening of inequalities rather than a factor that motivates key stakeholders in end-of-life care to concentrate their efforts in such environments.

## Conclusion

This study has demonstrated how the delivery of good quality end-of-life care in care homes requires an effective balance of external support, such as systems to access medication and syringe drivers, with internal resources, such as staff who are well trained and who work in a supportive culture in which they are able to make residents’ and their relatives’ needs and concerns their first priority. The mixed methods design has shed light on some critical factors that assist homes to manage some characteristic problems of access to external support: clinical leadership, clear understanding and vision about the need to improve end-of-life care, networking with GPs and other local staff and leverage of resources by one or two key external ‘supporters’ have been shown to be particularly important. In addition, the introduction of frameworks or pathways of care appears to assist staff to progress in terms of practice development and education in palliative and end-of-life care, but this needs further study.

The challenge of improving end-of-life care in care homes is usually described in terms of inadequacies in knowledge and training among care home staff. However, suggesting that training of care home staff will solve the issue of quality is a error of simplistic thinking.^40^ Rather, attention should in addition focus on challenging those discriminative attitudes, beliefs and practices in the wider system that contribute to the isolation of nursing homes and enhancing the ability of homes to demonstrate leadership in practice development. Although this exploratory study has provided some insights into the complex social structural network surrounding nursing homes, much more work is needed to enable integration of nursing homes into the wider systems of end-of-life care and to enable collaboration across organizational, institutional and funding boundaries, so that patients receive a better quality of end-of-life care regardless of the care setting in which they are located. Moreover, end-of-life care management in care homes should be integrated with and seen as an extension of chronic disease management and rehabilitation; attention to one will improve the other and is likely to result in improved quality of life, pain and symptom management for residents,^11^ regardless of any prognostic uncertainty about their status as ‘dying’.
